# Evaluating Diversity in Open Photoplethysmography Datasets: Protocol for a Systematic Review

**DOI:** 10.2196/73040

**Published:** 2025-10-01

**Authors:** Vedha Penmetcha, Lekaashree Rambabu, Brandon G Smith, Orla Mantle, Thomas Edmiston, Laura Hobbs, Shobhana Nagraj, Peter H Charlton, Tom Bashford

**Affiliations:** 1 International Health Systems Group, Department of Engineering, University of Cambridge, Cambridge, United Kingdom; 2 Department of Kinesiology, Wiess School of Natural Sciences, Rice University, 6360 Main St, Houston, TX, 77005, United States, 1 9133049350; 3 Department of Medicine, University of Cambridge, Cambridge, United Kingdom; 4 Department of Anaesthesia, Cambridge University Hospitals NHS Foundation Trust, Cambridge, United Kingdom; 5 Accelerate Programme for Scientific Discovery, Department of Computer Science and Technology, University of Cambridge, Cambridge, United Kingdom; 6 Cambridge Public Health Interdisciplinary Research Centre, University of Cambridge, Cambridge, United Kingdom; 7 NIHR Global Health Research Group on Acquired Brain and Spine Injury (ABSI), University of Cambridge, Cambridge, United Kingdom; 8 GKT School of Medical Education, King's College London, London, United Kingdom; 9 Department of Anaesthesia, East and North Hertfordshire Teaching NHS Trust, Stevenage, United Kingdom; 10 East London NHS Foundation Trust, , London, United Kingdom; 11 Department of Public Health and Primary Care, University of Cambridge, Cambridge, United Kingdom; 12 Nuffield Department of Women’s and Reproductive Health, University of Oxford, Oxford, United Kingdom

**Keywords:** photoplethysmography, PPG, open data, diversity, skin tone, bias, equity, fairness

## Abstract

**Background:**

Photoplethysmography (PPG) is an optical method for measuring blood volume changes in microcirculation through noninvasive photodetection. It has become a widespread and essential clinical tool, used in pulse oximeters and wearable devices. However, technical aspects of PPG make it susceptible to intrinsic bias, with the potential to adversely affect particular patient and consumer populations. Developments in PPG technology, increasingly driven by openly accessible datasets as opposed to de novo experimentation, have the potential to help monitor an array of physiological variables. However, some populations may be underrepresented in PPG datasets. We describe a protocol for a systematic review to assess the biases within open access PPG datasets.

**Objective:**

This review aims to evaluate the underlying reporting patterns and structure of openly accessible PPG datasets. We will provide insight into the measured biosignals and demographic variables included in the datasets in the hope of shedding light on what PPG data parameters are being used to develop medical devices. Therefore, we can elucidate current gaps and areas for improvement to reduce bias in medical device development.

**Methods:**

This review will be reported in accordance with the standard PRISMA (Preferred Reporting Items for Systematic Reviews and Meta-Analyses) guidelines. We will include primary studies that mention PPG and specifically reference openly accessible datasets since 2000. The datasets must contain physiological parameters such as heart rate, blood pressure, or respiratory rate, as well as the PPG waveform data, collected from humans. Searches will be conducted in literature databases and data repositories, including MedLine OVID, IEEE Xplore, Scopus, and PhysioNet. Studies will be evaluated in accordance with the Standing Together Initiative recommendations, which are urging for health care technologies supported by representative data. Biosignal and demographic variables will be extracted from the PPG datasets, with steps taken to harmonize and store this information. Statistical analysis will be performed, including descriptive statistics and the chi-square test for comparisons. Additional statistical analyses will be performed after data extraction is completed and the level of heterogeneity is characterized.

**Results:**

We will analyze the dataset diversity and the structural basis of PPG datasets. This includes statistically analyzing the demographic and biosignal variables in the datasets. By using statistical test fit for nominal variable comparisons, we will evaluate the frequencies of characteristics like the devices used, biosignals collected, clinical parameters, demographic characteristics, and geographic information. This systematic review is expected to be completed by September 2025. The screening and review of the articles is currently being conducted.

**Conclusions:**

This review will provide insight into the potential gaps of existing open access PPG datasets. It will inform future data collection and design of openly available PPG datasets for training medical devices, including wearables, to avoid perpetuating biases, allowing for application in diverse clinical settings.

## Introduction

Photoplethysmography (PPG) is an optical method for measuring blood volume changes in microcirculation, through noninvasive photodetection. It has become a widespread and essential tool, providing the sensing technology for pulse oximeters in clinical use and being widely used in consumer wearables such as smartwatches for heart rate monitoring. However, technical aspects of PPG make it susceptible to intrinsic bias, with the potential to adversely affect particular patient and consumer populations. Developments in PPG technology, increasingly driven by existing datasets as opposed to de novo experimentation, have the potential to help monitor an array of physiological variables with both clinical and wellness applications. However, these may disenfranchise those populations who are underrepresented in the existing training datasets.

PPG waveforms depict blood volume changes due to changes in absorption and reflection patterns detected by the photodetector [1]. PPG measurements are usually obtained by placing the device on the extremities of the body where the vascular bed is shallow and widespread [2]. These locations can include the wrist, fingers, toes, and ears. Through signal processing, and in some cases the use of different wavelengths of light, PPG can provide information on respiratory rate, blood pressure, sleep patterns, arterial stiffness, and blood oxygen saturation [3]. In addition, the periodicity of electrocardiogram and PPG waves can be correlated in terms of heart rate variability and arrhythmias, as the waveforms correlate to specific points within the cardiac cycle [4].

The 2 primary components of a PPG waveform consist of the pulsatile and the lower frequency portions. The pulsatile component directly correlates to the volumetric changes associated with the heart rate [5]. The lower frequency component, on the other hand, consists of the light absorbed by the tissue, veins, and blood, which correlates to volume capacity [5]. The optical measurements are usually taken at red or near-infrared wavelengths (625 nm-750 nm and 750 nm-1400 nm) or green wavelengths (500‐565 nm) [6]. These are commonly used because the red or near-infrared wavelengths penetrate up to 2 cm beyond the skin surface whereas the green penetrates up to 0.3 mm and allows for the differentiation between oxyhemoglobin and deoxyhemoglobin [7-9]. However, some PPG methods include the use of multiple wavelengths, including blue, green, and yellow light to improve the signal-to-noise ratio [10,11].

PPG, along with pulse oximetry, has limitations attributed to motion, ambient light, sensor contact, pulse pressure reduction, and disturbances in contact between the sensor and the skin. One key limitation that has recently been highlighted is that of inaccuracy across different skin tones [7,12,13]. Sjoding et al recently reported that black patients had almost 3 times the frequency of occult hypoxia–falsely elevated oxygen saturation levels–compared to their white counterparts [14]. Occult hypoxia can lead to higher risk of mortality and organ failure for darker-skinned patients and has been shown to reduce the provision of life-saving treatment modalities like supplemental oxygen and medications. This is clearly of concern, both due to the limitations of clinical devices but also the biases inherent in commercial health technology which systematically underserve select populations. The reduced performance of PPG-based technology on darker-skin-toned and obese patients necessitates further inclusion of demographic characteristics like skin tone, race or ethnicity, and even BMI in PPG datasets used to train medical devices, like pulse oximeters. Furthermore, as continuous clinical monitoring and the availability of wearable technology increases globally without correctional strategies, these biases will only worsen the performance of medical devices on diverse populations.

While the original experiments in pulse oximetry involved permissive hypoxia of test subjects, a number of current PPG developments are largely driven by training on available datasets. However, the biases within openly available datasets have not been described. This makes it difficult to understand their possible impact on both clinical and consumer technology. We describe a protocol for the first systematic review of which we are aware designed to assess the biases within currently open access PPG datasets. This has immediate value for those seeking to derive physiological data from PPG technology using training datasets. By focusing on openly accessible datasets and repositories, we will provide insight into the demographic characteristics of the data that is commonly used for a majority of existing PPG technology including wearables.

This review aims to evaluate the underlying reporting patterns and structure of openly accessible PPG datasets. We will provide insight into the measured biosignal and demographic variables included in datasets in hopes of shedding light on what PPG data parameters are being used to develop medical devices.

## Methods

### Overview

This review will be reported in accordance with the standard PRISMA (Preferred Reporting Items for Systematic Reviews and Meta-Analyses) guidelines. The study has been registered with PROSPERO (CRD42024564759) [15] before the initial literature search [16]. The full planned search strategy is provided in the Supplementary Material in Multimedia Appendix 1, which has been developed in close concordance with a medical librarian (IK and EE). This search strategy includes the databases and data repositories: MedLine OVID, IEEE, SCOPUS, Physionet, National Sleep Research Resource, UC Irvine Machine Learning Repository, and Borealis.

### Eligibility Criteria

We will include any peer-reviewed primary studies—observational and nonobservational—which use PPG datasets. This includes PPG datasets in published randomized controlled trials, nonrandomized trials, cross-sectional studies, cohort studies, and algorithmic models.

We will include studies published since 2000 to ensure the datasets are relevant to current technology developments. The year of 2000 was defined as the lower bound to ensure the datasets selected for analysis are aligned with contemporary medical device design, especially since there has been a rise in usage of PPG datasets to train wearable health technologies in the last 2 decades. In the early 2000s, significant global health milestones like the Millennium Development Goals were established, marking a period in time when critical frameworks and health care initiatives for promoting greater equity and diversity were implemented. We plan to include studies that mention PPG and specifically reference openly accessible datasets. Therefore, accounts will be created to access datasets as needed; however, any datasets that require a fee or monetary incentive to access the datasets will be deemed as not openly accessible. The datasets must contain physiological parameters such as heart rate, blood pressure, or respiratory rate as well as data describing the waveform, such as pulse wave or slope transit time. These terms have been chosen as they are indicators of parameters routinely collected in PPG datasets. The inclusion and exclusion criteria are outlined in Textbox 1. Included studies will be evaluated for the extent to which they include demographic characteristics and consider these characteristics.

Textbox 1. **Summary of inclusion and exclusion criteria.**

Inclusion criteria

- Openly accessible datasets
- Literature containing datasets years 2000-present
- Must contain photoplethysmography (PPG) signal and physiological parameters such as heart rate, blood pressure, or respiratory rate
- Human studies
- English and non-English articles, provided the non-English articles have datasets with English translation

Exclusion criteria

- Animal studies
- Lack of PPG signals
- Primary literature without reference to datasets
- Conference papers and abstracts

Datasets meeting the eligibility criteria will be included for analysis of demographic characteristics. Articles mentioning openly accessible datasets may be used to provide contextual information on the datasets, but only the datasets will be analyzed to ensure the primary objective of this review is focused on the reporting quality of openly accessible PPG datasets.

### Data Analysis

Following the initial literature search, all studies will be reviewed independently by two authors using systematic review software, Rayyan.ai [17,18]. Studies will initially be screened by title and abstract. The full-text articles will then undergo screening. Disagreements will be discussed with and resolved by a third author, a senior author with expertise in the topic. At each step, studies will be retrieved only if they meet the eligibility criteria.

#### Information Sources

Databases including Medline OVID, IEEE Xplore, and Scopus have been searched for articles including relevant datasets or mentionings of dataset repositories. The latest search was completed in December 2024. Following this literature search, specific dataset repositories including Physionet and GitHub will be searched to provide any additional openly accessible datasets relevant to the eligibility criteria and aim of the systematic review.

#### Risk of Bias

The studies will be assessed using the STANDING Together Initiative guidelines as a framework for evaluating the datasets, as there are no widely accepted tools for assessing the risk of bias of datasets [19-21]. The STANDING Together Initiative provides guidelines that address dataset diversity and inclusion to improve the transparency of dataset reporting [19]. Though these recommendations do not make up a definitive risk of bias tool, they provide content areas for analyzing the datasets, as they promote greater transparency in the reporting of datasets used for training and developing medical devices.

#### Synthesis of Results

After dataset extraction, a systematic analysis and summary of all included studies and datasets will be provided by describing their dataset characteristics and study outcomes. Due to the heterogeneity of the datasets included, the statistical analysis and summary will provide an overview of the current state of openly accessible PPG datasets and repositories. Details such as the devices, biosignals collected, clinical parameters (sample size), demographic characteristics (age, sex, race or ethnicity, skin tone, and BMI), and geographical information (country, health care setting, and dates) will provide insight into the dataset characteristics. We expect the need for stepwise data harmonization where variables included in the datasets will be extracted, compared, and stored in a CSV file format with standardized labeling. The analysis of the biosignal and demographic variables will be analyzed in R to allow for the creation of data visualizations and provide statistical comparisons of the variables included across the datasets. Quantitative analysis will allow this review to focus on the frequencies and counts of the measured biosignal and demographic data variables. This information will provide insight into the reporting quality of the datasets; however, the specific values or information collected within the dataset for each biosignal and demographic variable will not be analyzed, to ensure a broader understanding of dataset documentation.

Summary statistics will be provided based on the characteristics of the datasets. These will include descriptive statistics such as means, SD, and data visualizations depicting the distribution of demographic and geographical characteristics. Statistical analysis will be performed on the dataset characteristics, such as age, sex, race, ethnicity, skin tone, BMI, and other relevant parameters, including analysis on the frequency of the variables and other quantitative aspects extracted from the studies [21]. Raw counts of the characteristics will be visualized as percentages or proportions to depict the array of demographic variables reported in the PPG datasets. These characteristics will also be analyzed using statistical tests such as chi-square for frequency and independence [14]. These tests will provide insight into the differences in both the frequencies and the co-occurrence of demographic characteristics, respectively. Further analysis of the literature will contextualize why such differences may or may not be statistically significant. Visualizations such as histograms will include information on the mean ages, dataset sizes, and number of waveform characteristics reported. This analysis will provide insight into how datasets are structured and the amount of information reported in them to qualify potential reasons for statistically significant differences in dataset reporting. Additional statistical tests will be used, and information such as geographical or socioeconomic data will be displayed cartographically or in a heatmap to depict the geographical sources of PPG studies and datasets.

### Limitations and Implications

We predict that there will be heterogeneity in the data reporting quality and the terminology used to signify specific variables; therefore, it is important to use broad inclusion criteria to capture relevant articles.

We also anticipate significant heterogeneity of demographic characteristics. Jiang et al [19] reported that less than seven percent of physiological datasets, specifically those available on PhysioNet, include all 4 key demographic variables—age, sex, race, and ethnicity. Consequently, it is important to not include these variables in our inclusion or exclusion criteria to truly understand the level to which PPG datasets consider demographic aspects including variables such as age, sex, race, ethnicity, and other relevant parameters like skin tone or BMI. Understanding the extent to which demographic parameters are collected in PPG datasets will therefore shed light on what information is used in informing the development and deployment of healthcare technology. By collecting information on the structural aspects of the PPG datasets, we will provide a critical comprehension of the reporting quality of openly accessible datasets.

## Results

After the protocol was registered, the literature search was run in the databases IEEE, Scopus, and MedLine OVID in December 2024. The search yielded 1198 articles for initial screening. The openly accessible PPG datasets extracted from these repositories will be included in the analysis of demographic and biosignal data. The abstract screening phase has yielded 464 articles. The full-text screening is projected to be completed by July 2025. After which data analysis, detailed below, will be projected to be completed by September 2025.

## Discussion

### Principal Findings

Understanding the reporting quality of datasets used to develop health care technology is becoming increasingly important as the use of algorithmic models and processing to train medical devices grows. By analyzing openly accessible PPG datasets, we aim to provide a more comprehensive view of standard of data collection and usage for medical devices. Because of PPG’s long-standing usage in medical devices since the 1970 s and the wide range of biosignals that can be extracted from PPG data, it is vital to increase transparency in the demographic information collected in tangent to the biosignal data. The principal finding from this review will be to extract the different demographic and biosignal characteristics listed in openly accessible PPG datasets. We will provide summary statistics including frequencies, proportions, and distributions of the number and types of dataset characteristics. These summary statistics will be visualized in diagrams like histograms and box-whisker plots as well as tables. Any additional information related to characteristics such as the geographical location of the data collection or socioeconomic status will be portrayed in heatmaps to provide a general overview of additional social variables.

### Comparison to Previous Work

Wearables including smartwatches are commonly used to track personal health measures such as heart rate, sleep, and blood pressure. These commercially available medical devices often use PPG to take physiological measurements. However, PPG data collected by wearables have been observed to be affected by skin tone and higher adipose tissue due to obesity [12]. In combination, differences of up to 61% in signal intensity have been observed [12]. The Fitzpatrick scale is commonly used to assess for differences in skin tone, and while it provides an insight into the physiological signal values caused by greater melanin levels, it is subjective and treats skin tone as a categorical, rather than a continuous, variable [22]. This has limitations when considering the many underlying factors that result in the chromatic appearance of an individual’s skin tone, particularly if complicated by clinical conditions such as jaundice or hypoxia. Exploring the extent to which variables such as this are captured within existing datasets may be instructive in understanding their inherent ability to account for overall skin coloration.

Clearly, this same need to account for diversity in skin tone and clinical condition applies to clinical PPG studies and their resultant datasets. In a clinical setting, having erroneous oxygen saturation values or heart rate measurements can result in poorer clinical outcomes. There is evidence that the obese and those with greater melanin content in their skin endure worse health outcomes, with inaccurate monitoring a clear potential contributor [22]. Obesity can be attributed to physiological changes such as the reduction in trans-epidermal water loss and an increase in skin thickness due to adipose tissue can influence the light scattering patterns needed to obtain a PPG reading [22]. In addition to melanin content and obesity, other factors influencing perfusion and skin temperature can all affect PPG. When expanding PPG technology to low-resource settings, there is a need to account for diverse populations not only in terms of melanation but also factors such as obesity and conditions altering perfusion. Moreover, they may have a greater diversity of endemic clinical conditions including anemia, parasitemia, and malnutrition—whose effects on PPG have not been clearly described. Effective clinical PPG monitoring in these patients mandates an interpretation based on data from representative populations.

This understanding of demographic and biosignal variables is critical to presenting the current standard of reporting quality across openly accessible PPG datasets. In a recent review of PhysioNet datasets, a data repository with biosignal datasets, information regarding demographic and geographical information was presented to highlight the disparities and demographic gaps present in biosignal datasets [19]. This marks the analysis of openly accessible PPG datasets relevant and important for promoting transparency and equity in medical device development, as PPG is a widely accepted technology growing in popularity in health care. The aims of this systematic review mirror those of the review by Jiang et al [19], but take a broader look at openly accessible PPG datasets across multiple databases and repositories.

### Strengths and Limitations

Providing a detailed summary of the demographic and biosignal variables in openly available datasets, we aim to promote a more comprehensive understanding of reporting standards in existing PPG datasets. This review will provide a structured overview of the current gaps and sources of bias in developing PPG-based medical devices. Transparency in dataset reporting will highlight the need for greater diversity and promote an increased range of demographic characteristics collected in openly available PPG datasets. However, we do expect limitations such as heterogeneity in the datasets, especially as it comes to which characteristics are collected and included in the datasets. We also anticipate challenges in gaining access to certain datasets as we will likely have to go through repositories or databases that require account creation or multiple steps in gaining access to the PPG databases. These limitations will be mitigated by maintaining clear documentation of the characteristics extracted from the datasets being analyzed and data harmonization. Also, accounts will be created as needed to ensure adequate access is gained to review the dataset characteristics.

### Future Directions

In this systematic review, we will analyze the structural basis of PPG datasets and critically evaluate the different variables included in the datasets. This analysis will highlight the reporting quality of openly accessible PPG datasets. By critically appraising the dataset diversity and reporting quality, we can highlight the gaps present in current PPG data to inform future collection and design of medical devices including wearables to be more inclusive to avoid perpetuating biases. In addition, by looking at the structure of PPG datasets and their representativeness, we can also provide insight into the gaps that may be present in the context of PPG applications in low-resource settings. The findings can promote awareness of sources of bias in openly accessible PPG datasets, informing future data collection and modulation when training PPG-based medical technology. With the synthesis of this information and data analysis, we hope that medical device developers would consider dataset level biases and their implications on human health. We hope that these considerations would push toward greater transparency in PPG data collection and extraction of demographic and biosignal characteristics to ensure users are informed of the design and development of medical devices and avenues for promoting fairness. This review, therefore, aims to address disparities in openly available PPG datasets, the downstream effects of these disparities, and potential avenues for greater equity in PPG dataset creation and usage.

## Supplementary material

10.2196/73040 Multimedia Appendix 1 Search strategy.

10.2196/73040 Checklist 1 PRISMA-P checklist.

## Abbreviations

PPG: Photoplethysmography
PRISMA: Preferred Reporting Items for Systematic Reviews and Meta-Analyses
