# Supplementary material for: Evaluating Diversity in Open Photoplethysmography Datasets: Protocol for a Systematic Review
Source: JMIR Res Protoc. 2025 Oct 1;14:e73040. doi: 10.2196/73040 (PMC12488168; doi:10.2196/73040)
Supplement: Multimedia Appendix 1 [file resprot-v14-e73040-s001.docx]

Search Strategy:

1            exp Photoplethysmography/

2            (photoplethysmogra* or ppg or polysomnogra*).ti,ab,kw,kf.

3            1 or 2

4            (data set or data sets or dataset*).ti,ab,kw,kf.

5            exp Dataset/

6            4 or 5

7            3 and 6

8            ((physiological adj (signal* or monitor*)) or blood pressure or pulse or heart rate* or volume pulse).ti,ab,kw,kf.

9            exp Blood Pressure/

10         exp Pulse/

11         exp Heart Rate/

12         exp Monitoring, Physiologic/

13         8 or 9 or 10 or 11 or 12

14         exp Wearable Electronic Devices/

15         (non-invasive medical technolog* or wearable*).ti,ab,kw,kf.

16         14 or 15

18         3 and 6 and (13 or 16)
